# Ferric Hydrogensulfate [Fe(HSO_4_)_3_] As a Reusable Heterogeneous Catalyst for the Synthesis of 5-Substituted-1*H*-Tetrazoles and Amides

**DOI:** 10.5402/2011/195850

**Published:** 2011-03-29

**Authors:** Hossein Eshghi, Seyed Mohammad Seyedi, Elaheh Rahimi Zarei

**Affiliations:** Department of Chemistry, Faculty of Science, Ferdowsi University of Mashhad, P.O. Box 91775-1436, Mashhad, Iran

## Abstract

Ferric hydrogensulfate catalyzed the synthesis of 5-substituted 1*H*-tetrazoles via [2 + 3] cycloaddition of nitriles and sodium azide. This method has the advantages of high yields, simple methodology, and easy workup. The catalyst can be recovered by simple filtration and reused delivering good yields. Also, ferric hydrogensulfate catalyzed the hydrolysis of nitriles to primary amides under aqueous conditions. Various aliphatic and aromatic nitriles converted to the corresponding amides in good yields without any contamination with carboxylic acids.

## 1. Introduction

The literature on tetrazole chemistry has been expanded rapidly. This is mainly as a result of the role played by tetrazoles in coordination chemistry as ligands, in medicinal chemistry as stable surrogates for carboxylic acids, and in materials applications, including explosives, rocket propellants, and agriculture [1–3]. Tetrazoles can be used as isosteric replacements for carboxylic acids in drug design [1]. An advantage of tetrazolic acids over carboxylic acids is that they are resistant to many biological metabolic degradation pathways [2]. The most convenient method of synthesizing tetrazoles is the addition of azide ions to nitriles. Earlier reported methods for the synthesis of 5-substituted tetrazoles suffer from drawbacks such as the use of strong Lewis acids, or expensive and toxic metals, and the *in situ *generated hydrazoic acid which is highly toxic and explosive [4]. Several syntheses of 5-substituted tetrazoles have been reported through the [2 + 3] cycloaddition of nitriles using NaN_3_ or TMSN_3_ in the presence of catalysts such as ZnBr_2_ [5], ZnCl_2_ [6], FeCl_3_-SiO_2_ [7], TBAF [8], Zn/Al hydrotalcite [9], ZnO [10], and Cu_2_O [11]. 

On the other hand, several syntheses of primary amides from nitriles have been reported using NaBO_3_/MW [12], ZnCl_2_/MW [13], ruthenium complex/sealed tube [14], and ZnX_2_/ketoxime combination [15]. However, most of the reported methods have not been proven to be general or practical in scope because of harsh condition or expensive catalysts. 

**Scheme 1:**
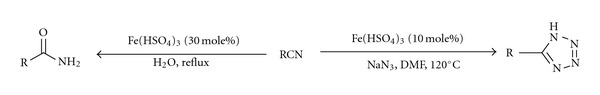
Conversion of nitriles to 1*H*-tetrazoles and amides using ferric hydrogensulfate.

In continuation of our recent work on applications of heterogeneous reagents for the development of synthetic methodologies [16–19], we wish to report a new protocol for the preparation of 5-substituted-1*H*-tetrazoles from a wide variety of nitriles using Fe(HSO_4_)_3_ as a solid acid catalyst (Scheme 1). Also, we introduce ferric hydrogensulfate as an efficient catalyst for the hydrolysis of nitriles to primary amides under aqueous conditions (Scheme 1). 

## 2. Experimental

Chemicals were either prepared in our laboratories or purchased from Merck, Fluka, and Aldrich Chemical companies. All yields refer to isolated products. The reactions were monitored by thin-layer chromatography carried out on silica plates. The products were characterized by comparison of their physical data with authentic samples or by their spectral data. IR spectra were recorded on a Shimadzu-IR 470 spectrophotometer. ^1^H NMR spectra were recorded on Bruker 100-MHz and 500 MHz spectrometer in DMSO as the solvent and TMS as internal standard. Ferric hydrogensulfate was prepared according to previously reported procedure [16, 17].

### 2.1. General Procedure to Synthesis of 5-(Substituted)-1*H*-Tetrazoles

Ferric hydrogensulfate (0.2 mmoL) was added to nitrile (2 mmoL), sodium azide (0.2 g, 3 mmoL), and distilled dimethylformamide (6 mL), and the mixture was stirred at 120°C for 20 h (Table 2). After completion of the reaction (as indicated by TLC), the catalyst was removed by filtration and the filtrate was treated with ethyl acetate (35 mL) and 4 N HCl (20 mL) and stirred vigorously. The resultant organic layer was separated and the aqueous layer was extracted with ethyl acetate (25 mL). The combined organic layer was washed with water (8 mL) and concentrated to give a crude product. Column chromatography using silica gel gave pure product in high yields. The pure product was characterized and identified by their melting point, IR, ^1^H NMR, and elemental analysis and compared with those reported.

### 2.2. Selected Product Characterization Data

5-(3,5-Dimethoxyphenyl)-1H-tetrazole (Table 2, entry 8): ^1^H NMR (100 MHz, CD_3_COCD_3_): *δ* = 8.5 (b, 1H, NH), 7.2 (d, *J* = 2 Hz, 2H, 2,6-H), 6.65 (t, *J* = 1.5 Hz, 1H, 4-H), 3.9 (s, 6H, CH_3_O); IR (KBr) *υ* = 3450 (NH) cm^−1^. Anal. Calcd for C_9_H_10_N_4_O_2_: C, 52.42; H, 4.89; N, 27.17; O, 15.52. Found: C, 52.95; H, 4.53; N, 26.98. 

 5-(Phenanthren-9-yl)-1H-tetrazole (Table 2, entry 9): ^1^H NMR (100 MHz, CD_3_COCD_3_): *δ* = 8.95 (dd, *J* = 8, 2 Hz, 1H, 4-H), 8.8 (b, 1H, NH), 8.65 (dd, *J* = 8, 2 Hz, 1H, 3-H), 8.35 (s, 1H, 10-H), 8.1 (dd, *J* = 8, 2 Hz, 1H, 5-H), 7.1–7.9 (m, 5H, 1,2,6,7,8-H); IR (KBr) *υ* = 3450, 3150 (NH) cm^−1^. Anal. Calcd for C_15_H_10_N_4_: C, 73.16; H, 4.09; N, 22.75; Found: C, 73.63; H, 3.97; N, 22.14.

### 2.3. General Procedure to Preparation of Primary Amides from Nitriles

Ferric hydrogensulfate (30 mole %) was added to a solution of nitrile (4 mmoL) in water (10 mL) and refluxed for 48 h. After completion of the reaction (as indicated by TLC), the reaction mixture was cooled to room temperature and neutralized with sodium hydroxide solution (4 N) to pH 7 carefully. The reaction mixture was filtered and extracted with ethyl acetate (2 × 20 mL). The organic layer dried over sodium sulfate and evaporated. The crystalline amide was obtained after recrystallization from H_2_O-EtOH. The products were obtained in 45–72% yields. All the products are known compounds, and the spectral data and melting points were identical to those reported in the literature.

 Acrylamide (Table 3, entry 10): ^1^H NMR (500 MHz, DMSO-*d*6): *δ* = 7.5 (b, 1H, NH), 7.1 (b, 1H, NH), 6.18 (dd, *J* = 16, 10 Hz, 1H, *α*-H), 6.07 (dd, *J* = 16, 2.2 Hz, 1H, *β*_trans_-H), 5.58 (dd, *J* = 10, 2.2 Hz, 1H, *β*_cis_-H); IR (KBr) *υ* = 3370, 3320 (NH_2_), 1650 (CO) cm^−1^.

## 3. Results and Discussion

First, we optimized the amount of Fe(HSO_4_)_3_ catalyst required for the preparation of tetrazoles in the reaction between benzonitrile and sodium azide (Table 1). Water was not a suitable solvent for this reaction. The optimum amount of Fe(HSO_4_)_3_ was found to be 10 mole % in the presence of nitrile (2 mmoL) and sodium azide (3 mmoL) in DMF (6 mL). The best result (96% isolated yield) was obtained after 18 h reflux in DMF (Table 1, entry 2), while, increasing the reaction time to 24 h does not improved the isolated yield (Table 1, entry 3). 

We next examined the scope and generality of the Fe(HSO_4_)_3_-promoted [2+3] cycloaddition reaction to form 5-substituted 1*H*-tetrazoles, and the results are summarized in Table 2. The nature of the substituent on the benzonitrile did not affect the reaction time (Table 2, entries 3, 4, and 7). However, 4-hydroxybenzonitrile and aliphatic nitriles needed long reaction times (Table 2, entries 5, 10, and 11). 

The Fe(HSO_4_)_3_ catalyst was recovered from the reaction mixture by simple filtration and was purified by washing the solid residue with CH_2_Cl_2_. The recovered catalyst was used four times without any loss of activity (Table 2, entry 1). 

The products were characterized by IR and ^1^H-NMR spectroscopy and their melting points compared with those of authentic samples. The disappearance of one strong and sharp absorption band (CN stretching band) and the appearance of an NH stretching band in the IR spectra were evidence for the formation of 5-substituted 1*H*-tetrazoles. 

To the best of our knowledge only one example of conversion of nitrile to primary amides in the presence of ferric salts was reported in the literature by using Fe(NO_3_)_3_·9H_2_O in sealed tube condition at 125°C [20].

In a typical procedure the ferric hydrogensulfate (30 mole %) and nitrile (4 mmoL) were dissolved in water (10 mL) and refluxed for 48 h. After usual workup by neutralization of the reaction mixture to pH 7 and extraction with ethyl acetate, the products were obtained in 45–72% yields. The results are shown in Table 3. In general, aliphatic and aromatic nitriles were successfully converted into amides. Two industrially important amides, acryl amide and methacrylamide were obtained in 69% and 60% yields respectively, from the corresponding nitriles. 

The products were characterized by IR and ^1^H-NMR spectroscopy and their melting points compared with those of authentic samples. The disappearance of one strong and sharp absorption band (CN stretching band) and the appearance of two NH_2_ stretching bands at 3370 and 3320 cm^−1^ and carboxamide stretching at 1650 cm^−1^ in the IR spectra were evidence for the formation of primary amides. In the ^1^H-NMR spectrum of acrylamide, three doublets were observed for three vinylic protons at 5.57, 6.07, and 6.17 ppm showing the expected vicinal and germinal splitting. The NH_2_ group appears as two singlets at 7.1 and 7.5 ppm due to amidic resonance. 

The mechanism of this selective transformation is not clear. We assume that ferric hydrogensulfate acts as a Lewis or Bronsted acid making the nitrile more susceptible to nucleophilic addition. Perhaps an efficient coordination of the amide intermediate with the ferric ion stops the hydrolysis at the amide stage until workup. The observed stoichiometry of the reaction (nitrile:ferric ion: 3 : 1) supports this explanation.

## 4. Conclusion

In conclusion, we have developed a novel and highly efficient method for the synthesis of 5-substituted 1*H*-tetrazoles by treatment of nitriles with sodium azide in the presence of Fe(HSO_4_)_3_ as catalyst. The significant advantages of this methodology are high yields, a simple workup procedure, and easy preparation and handling of the catalyst. The catalyst can be recovered by filtration and reused. Hydrolysis of nitriles to primary amides is another feature of this catalyst under aqueous conditions.

## Figures and Tables

**Table 1:** Preparation of 5-phenyl-1*H*-tetrazoles using varying amounts of Fe(HSO_4_)_3_ under reflux conditions.

Entry	Solvent	Mole ratio of nitrile : azide	Fe(HSO_4_)_3_ mole %	Time (h)	Yield^a^ %
1	DMF	1 : 1.5	10	12	70
2	DMF	1 : 1.5	10	18	96
3	DMF	1 : 1.5	10	24	96.5
4	DMF	1 : 1.5	20	12	91
5	DMF	1 : 1.5	5	12	49
6	DMF	1 : 1.5	0	18	0
7	DMF	1 : 1	10	18	72
8	H_2_O	1 : 1.5	10	24	20
9	THF	1 : 1.5	10	24	5

(a) Isolated yields.

**Table 2:** Preparation of 5-subsitituted-1*H*-tetrazoles in the presence of Fe(HSO_4_)_3_.

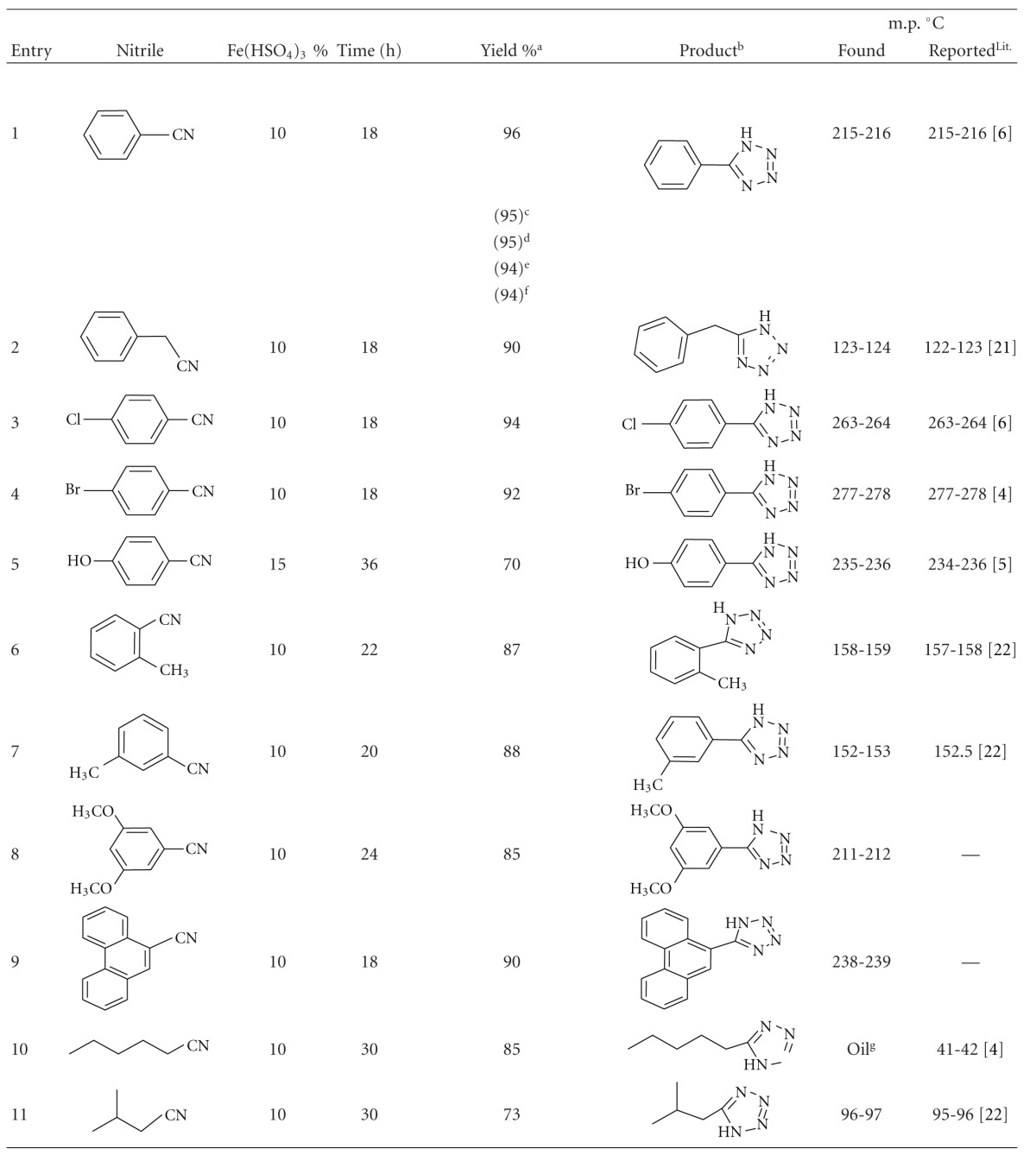

(a) Isolated yields. (b) Recrystallized from CH_2_Cl_2_-*n*-hexane. ((c)–(f)) Yields in parentheses refer to reusability of the recovered catalyst in new runs. (g) Purified only with column chromatography using CH_2_Cl_2_-*n*-hexane.

**Table 3:** Preparation of primary amides from nitriles in the presence of Fe(HSO_4_)_3_.

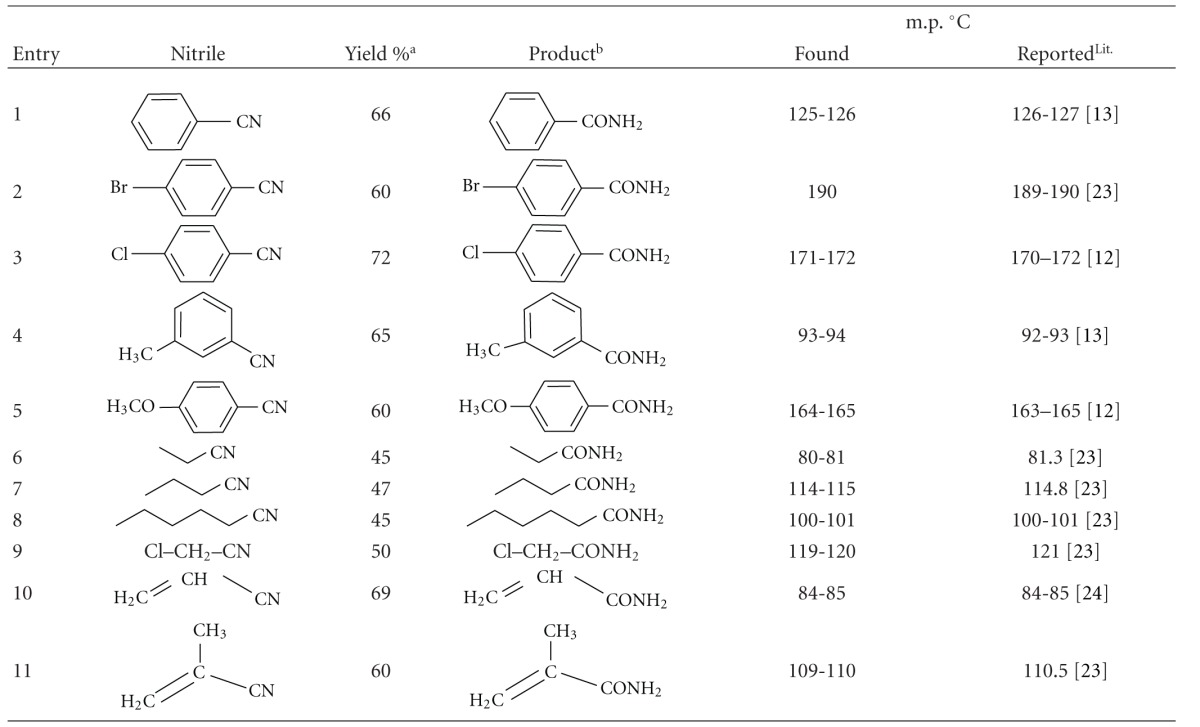

(a) Yields refer to the isolated products. (b) The products were recrystallized from H_2_O-EtOH and characterized by IR and ^1^H-NMR spectroscopy, and also their melting points are compared with authentic samples.
